# Compositional Variation and Bioactivity of the Leaf Essential Oil of *Montanoa guatemalensis* from Monteverde, Costa Rica: A Preliminary Investigation

**DOI:** 10.3390/medicines2040331

**Published:** 2015-11-24

**Authors:** Victoria D. Flatt, Carlos R. Campos, Maria P. Kraemer, Brittany A. Bailey, Prabodh Satyal, William N. Setzer

**Affiliations:** Department of Chemistry, University of Alabama in Huntsville, Huntsville, AL 35899, USA; E-Mails: flattvd@gmail.com (V.D.F.); campocr2013@gmail.com (C.R.C.); maria.c.palazzo@vanderbilt.edu (M.P.K.); baileybr@umich.edu (B.A.B.); ps0013@uah.edu (P.S.)

**Keywords:** essential oil composition, cyclocolorenone, selinene, muuroladiene, cadinene, limonene, pinene, cytotoxicity

## Abstract

Background: *Montanoa guatemalensis* is a small to medium-sized tree in the Asteraceae that grows in Central America from Mexico south through Costa Rica. There have been no previous investigations on the essential oil of this tree. Methods: The leaf essential oils of *M. guatemalensis* were obtained from different individual trees growing in Monteverde, Costa Rica, in two different years, and were analyzed by gas chromatography—mass spectrometry. Results: The leaf oils from 2008 were rich in sesquiterpenoids, dominated by α-selinene, β-selinene, and cyclocolorenone, with lesser amounts of the monoterpenes α-pinene and limonene. In contrast, the samples from 2009 showed no α- or β-selinene, but large concentrations of *trans*-muurola-4(14),5-diene, β-cadinene, and cyclocolorenone, along with greater concentrations of α-pinene and limonene. The leaf oils were screened for cytotoxic and antimicrobial activities and did show selective cytotoxic activity on MDA-MB-231 breast tumor cells. Conclusion: *M. guatemalensis* leaf oil, rich in cyclocolorenone, α-selinene, and β-selinene, showed selective *in vitro* cytotoxic activity to MDA-MB-231 cells. The plant may be a good source of cyclocolorenone.

## 1. Introduction

*Montanoa guatemalensis* B.L. Rob. & Greenm. (Asteraceae), “tubú”, is a small to medium tree (3–15 m) that is found in Central America from Mexico through Costa Rica. The leaves are around 13 × 17 cm and have 2–4 shallow lobes. The flowers have large heads (5 cm diameter) with white ray flowers (2.5 cm long) and orange inner disk flowers [1]. This tree has been planted as windbreaks in Monteverde [2]. Germacranolide sesquiterpenoids have been isolated from *M. guatemalensis* [3], but to our knowledge, the volatile composition of this tree has not been previously investigated. In this work, we present the leaf essential oil composition of *M. guatemalensis*.

## 2. Experimental Section

### 2.1. Plant Material

Leaves of *Montanoa guatemalensis* were collected from two different mature trees (tree A and tree B, flowering stage) on 3 May 2008, and two different mature trees (tree C and tree D, flowering stage) on 7 May 2009, from the property of Hotel El Bosque, Monteverde, Costa Rica (10.3059 N, 84.8144 W, 1380 m above sea level). The plant was identified by William Haber, and a voucher specimen (Haber 425) has been deposited in the herbarium of the Missouri Botanical Garden. The fresh leaves were chopped and hydrodistilled using a Likens-Nickerson apparatus with continuous extraction with CHCl_3_ to give the yellow essential oils (Table 1).

**Table 1:** Yields of leaf essential oils from *Montanoa guatemalensis*.

Sample	Mass Fresh Leaves	Mass Essential Oil	Yield
2008, tree A	46.3 g	188 mg	0.41%
2008, tree B	34.0 g	160 mg	0.47%
2009, tree C	122.1 g	525 mg	0.43%
2009, tree D	192.1 g	791 mg	0.41%

### 2.2. Gas Chromatographic—Mass Spectral Analysis

A gas chromatographic—mass spectral analysis was performed on the essential oils of *M*. *guatemalensis* using an Agilent 6890 GC with Agilent 5973 mass selective detector (Santa Clara, CA, USA) (EIMS, electron energy = 70 eV, scan range = 45–400 amu, and scan rate = 3.99 scans/s), and a fused silica capillary column (HP-5ms, 30 m × 0.25 mm) coated with 5% phenyl-polymethylsiloxane (0.25 mm phase thickness). The carrier gas was helium with a flow rate of 1 mL/min, and the injection temperature was 200 °C. The oven temperature was programmed to initially hold for 10 min at 40° C, then ramp to 200 °C at 3 °C/min and finally to 220 °C at 2 °C/min. The interface temperature was 280 °C. A 1% *w*/*v* solution of each sample in CHCl_3_ was prepared, and 1 μL was injected using a splitless injection technique.

Identification of the oil components was based on their retention indices determined by reference to a homologous series of *n*-alkanes, and by comparison of their mass spectral fragmentation patterns with those available in commercial libraries [4,5] as well as our own in-house library [6]. The percentages of each component are reported as raw percentages based on total ion current without standardization. The chemical compositions of the *M*. *guatemalensis* leaf oils are summarized in Table 2.

**Table 2:** Chemical compositions of the leaf essential oils of *Montanoa guatemalensis*.

RI ^a^	Compound	2008		2009	
		Tree A	Tree B	Tree C	Tree D
923	Artemisia triene	0.69	0.13	-	-
939	α-Pinene	3.18	3.78	6.91	8.56
958	Camphene	0.76	0.68	-	2.18
980	Sabinene	0.32	0.07	-	-
982	β-Pinene	0.74	0.93	1.81	3.71
990	Myrcene	0.12	0.12	0.10	0.27
1007	*iso*-Sylvestrene	1.65	0.48	-	1.84
1030	Limonene	8.25	8.05	21.31	12.15
1040	(*Z*)-β-Ocimene	tr ^b^	tr	-	-
1051	(*E*)-β-Ocimene	1.42	0.96	2.42	1.72
1063	Artemisia ketone	-	-	1.77	-
1075	*cis*-Linalool oxide (furanoid)	0.52	tr	-	-
1086	Artemisia alcohol	0.05	tr	1.67	0.67
1106	Linalool	-	-	0.14	-
1165	Borneol	0.53	0.08	-	-
1178	Artemisyl acetate	0.82	0.92	9.79	1.20
1282	Bornyl acetate	0.06	tr	-	-
1340	δ-Elemene	2.27	1.45	1.43	1.40
1349	α-Cubebene	-	tr	-	-
1365	α-Ylangene	-	tr	-	-
1371	Unidentified ^c^	0.19	-	1.28	-
1376	α-Copaene	0.21	0.32	-	1.32
1384	α-Bourbonene	-	tr	-	-
1391	β-Elemene	0.16	0.10	-	-
1391	β-Cubebene	-	0.12	0.11	0.20
1408	α-Gurjunene	0.09	-	-	-
1420	(*E*)-Caryophyllene	3.39	1.39	2.46	2.14
1429	β-Gurjunene (=Calarene)	0.07	0.08	-	-
1436	γ-Elemene	0.26	0.39	0.30	0.42
1439	α-Guaiene	0.07	0.10	-	-
1444	6,9-Guaiadiene	0.26	0.19	0.13	0.06
1447	9-*epi*-(*E*)-Caryophyllene	tr	tr	-	-
1454	α-Humulene	0.75	0.59	0.82	0.59
1460	*allo*-Aromadendrene	0.26	0.12	-	-
1493	β-Selinene	8.69	14.77	-	-
1494	*trans*-Muurola-4(14),5-diene	-	-	9.49	16.81
1500	Bicyclogermacrene	2.03	2.10	1.40	3.05
1516	α-Selinene	21.15	15.90	-	-
1518	β-Cadinene	-	0.20	13.26	11.10
1522	δ-Cadinene	0.86	0.94	-	0.42
1545	Selina-3,7(11)-diene	3.63	4.54	3.55	7.96
1548	γ-Vetivenene	1.79	2.09	-	-
1561	Germacrene B	3.49	4.20	1.98	2.48
1570	Palustrol	-	-	-	0.72
1582	Zierone	0.86	0.52	0.30	-
1590	Khusimone	0.20	0.13	0.39	0.35
1603	Guaiol	-	-	-	0.65
1629	Unidentified ^d^	2.29	2.62	1.00	2.92
1642	Selina-3,11-dien-6α-ol	0.36	0.33	0.14	-
1653	Atractylone	0.70	0.56	0.47	0.63
1667	Unidentified ^e^	0.79	0.55	0.14	-
1688	α-Bisabolol	0.50	0.19	-	0.07
1718	*neo*-Cyclocolorenone	0.09	tr	-	-
1743	Cyclocolorenone	23.82	27.54	14.49	15.61
1762	Methyl 3,9,11-guaiatrien-12-oate	0.38	0.18	0.51	-
1833	*iso*-Cyclocolorenone	0.17	0.06	-	-
1950	Geranyl-α-terpinene	0.13	-	-	-
2006	Geranyl-*p*-cymene	0.17	0.34	-	-
-	Monoterpenoids	19.40	16.53	45.92	32.31
-	Sesquiterpenoids	79.77	82.27	53.64	68.90
-	Total Identified	95.90	95.64	97.14	98.28

^a^ RI = Retention Index determined in reference to a homologous series of *n*-alkanes on an HP-5ms column; ^b^ tr = “trace” (<0.05%); ^c^ MS (EI, m/e): 170(5), 169(24), 136(16), 121(79), 107(37), 105(41), 93(100), 91(73), 85(90), 79(59), 77(49), 74(60), 57(94); ^d^ MS (EI, m/e): 204(33), 202(100), 187(71), 173(19), 161(21), 159(51), 145(53), 131(61), 119(36), 105(54), 93(55), 91(71), 79(43), 77(36); ^e^ MS (EI, m/e): 218(100), 203(23), 175(17), 159(30), 149(29), 133(28), 119(25), 108(40), 105(35), 95(51), 93(56), 91(51), 79(43), 77(34), 67(25), 53(19).

### 2.3. Antibacterial Screening

The *M. guatemalensis* leaf oils were screened for antibacterial activity against *Bacillus cereus* (American Type Culture Collection, Manassas, VA, USA, ATCC No. 14579), *Staphylococcus aureus* (ATCC No. 29213), and *Escherichia coli* (ATCC No. 10798). Minimum inhibitory concentrations (MICs) were determined using the microbroth dilution technique [7]. Dilutions of the crude extracts were prepared in cation-adjusted Mueller-Hinton broth (Fisher Scientific, Pittsburgh, PA, USA) (CAMHB) beginning with 50 μL of 1% *w*/*w* solutions of crude extracts in dimethylsulfoxide (Sigma-Aldrich, St. Louis, MO, USA) (DMSO) plus 50 μL CAMHB. The extract solutions were serially diluted (1:1) in CAMHB in 96-well plates. Organisms at a concentration of approximately 1.5 × 10^8^ colony-forming units (CFU)/mL were added to each well. Plates were incubated at 37 °C for 24 h; the final minimum inhibitory concentration (MIC) was determined as the lowest concentration without turbidity. Geneticin^®^ (Sigma-Aldrich, St. Louis, MO, USA) was used as a positive antibiotic control; DMSO was used as a negative control.

### 2.4. Cytotoxicity Screening

Human MDA-MB-231 breast adenocarcinoma cells (ATCC No. HTB-26) [8] were grown in an air environment at 37 °C in Leibovitz’s L-15 medium (Fisher Scientific, Pittsburgh, PA, USA) with l-glutamine, supplemented with 10% fetal bovine serum, 100,000 units penicillin and 10.0 mg streptomycin per liter of medium, and buffered with 30 mM (4-(2-hydroxyethyl)-1-piperazineethanesulfonic acid ) (Hepes, Fisher Scientific, Pitsburgh, PA, USA), pH 7.35. Human Hs578T breast ductal carcinoma cells (ATCC No. HTB-129) [9] were grown in a 3% CO_2_ environment at 37 °C in Dulbecco’s Modified Eagle Medium (DMEM) (Fisher Scientific, Pittsburgh, PA, USA) with 4500 mg glucose per liter of medium, supplemented with 10% fetal bovine serum, 10 μg bovine insulin, 100,000 units penicillin and 10.0 mg streptomycin per liter of medium, and buffered with 44 mM NaHCO_3_, pH 7.35.

MDA-MB-231 cells were plated into 96-well cell culture plates, at 2.6 × 10^4^ cells per well, and Hs578T cells at 1.0 × 10^5^ cells per well. The volume in each well was 100 μL for all cell types. After 48 h, supernatant fluid was removed by suction and replaced with 100 μL growth medium containing 1.0 μL of DMSO solution of essential oils (1% *w*/*w* in DMSO), giving a final concentration of 100 μg/mL for each oil. Solutions were added to wells in four replicates. Medium controls and DMSO controls (10 μL DMSO/mL) were used. Tingenone (100 μg/mL) was used as a positive control [10]. After the addition of compounds, plates were incubated for 48 h at 37 °C; medium was then removed by suction, and 100 μL of fresh medium was added to each well. In order to establish percent kill rates, the 3-(4,5-dimethylthiazol-2-yl)-2,5-diphenyltetrazolium bromide (MTT, Sigma-Aldrich, St. Louis, MO, USA) assay for cell viability was carried out [11]. After colorimetric readings were recorded (using a Molecular Devices (Sunnyvale, CA, USA) SpectraMAX Plus microplate reader, 570 nm), average absorbances, standard deviations, and percent kill ratios (%kill_oil_/%kill_DMSO_) were calculated. Cytotoxic activities of the essential oils are summarized in Table 3.

**Table 3:** Bioactivity screening of the leaf essential oils of *Montanoa guatemalensis*.

Essential Oil Sample	Antibacterial (MIC, μg/mL)			Cytotoxicity (% Kill at 100 μg/mL)	
	*B. cereus*	*S. aureus*	*E. coli*	MDA-MB-231	Hs578T
2008, tree A	313	1250	2500	100	6.5 ± 8.8
2008, tree B	625	1250	2500	100	0
2009, tree C	313	1250	2500	66.3 ± 5.6	0
2009, tree D	313	1250	2500	21.9 ± 3.5	0

## 3. Results and Discussion

The leaves from two different mature trees were collected in 2008 and from two different trees in 2009. The leaves were hydrodistilled to give pale yellow essential oils (0.41%–0.47% yield), which were analyzed by gas chromatography—mass spectrometry (Table 2). The major components in the leaf oils from 2008 were cyclocolorenone (23.8% and 27.5%), α-selinene (21.2% and 15.9%), β-selinene (8.7% and 14.8%), and limonene (8.3% and 8.1%). The 2008 leaf oils were dominated by sesquiterpenoids (79.8% and 82.3%). Although qualitatively similar, there were some important differences in the leaf oils from 2009: Sesquiterpenoid concentrations were lower (53.6% and 68.9%) with correspondingly increased monoterpenoids (45.9% and 32.3%) in 2009; neither α-selinene nor β-selinene were detected in the 2009 leaf oils; there were large concentrations of *trans*-muurola-4(14),5-diene (9.5% and 16.8%), which were not detected in 2008, and large concentrations of β-cadinene (13.3% and 11.1%), which was seen in only one tree from 2008 in small (0.2%) quantity. Other major components in the 2009 leaf oils were cyclocolorenone (14.5% and 15.6%), limonene (21.3% and 12.2%), and α-pinene (6.9% and 8.6%).

Cyclocolorenone has been observed in several species of Asteraceae. The compound was found to be a major component of the essential oils of *Solidago gigantea* (Asteraceae) (32.8%) [12,13], *Solidago canadensis* (Asteraceae) (38%) [14], as well as *Drimys braziliensis* (Winteraceae) (18.2%) [15]. The compound has also been found in the essential oils of *Acritopappus confertus* (Asteraceae) [16] and *Vernonia brasiliana* (Asteraceae) [17], as well as *Ledum palustre* (Ericaceae) [18] and *Eugenia copacabanensis* (Myrtaceae) [19].

Several Asteraceae species have been shown to be rich in α-selinene, including *Baccharis crispa*, *Baccharis milleflora* [20], and *Tridax procumbens* [21]. β-Selinene has been observed in *B. milleflora* [20] and *Encelia farinosa* [22]. The leaf oils of *Heterothalamus alienus* (Asteraceae) from Argentina [23], *Tagetes minuta* (Asteraceae) from Kenya [24], *Blumea perrottetiana* (Asteraceae) from Nigeria [25], and *Clibadium leiocarpum* from Costa Rica [26] have all shown *trans-*muurola-4(15),5-diene in their compositions.

Comparison of the leaf oils from *M. guatemalensis* with those from other *Montanoa* species shows little similarity in composition. The major components in the leaf oil from *M. grandiflora* from Mexico were α-pinene (4.3%), β-phellandrene (4.2%), limonene (2.4%), citronellal (2.8%), and guayacol (2.0%) [27], while *M. tomentosa* leaf oil from Mexico was dominated by bornyl acetate (26.3%), (*E*)-caryophyllene (12.5%), β-cubebene (24.0%), limonene (4.9%), and borneol (4.1%) [28]. Headspace analysis of *M. tomentosa* leaves showed a predominance of monoterpenes, α-pinene (15.9%), α-thujene (10.4%), santolina triene (4.6%), sabinene (39.5%), limonene (3.7%), and γ-terpinene (5.1%) [29], but volatiles from the glandular trichomes of *M. tomentosa* were dominated by the sesquiterpenoids valencene (25.3%–45.0%) and β-eudesmol (27.2%–56.1%) [30].

The leaf oils of *M. guatemalensis* were screened for antibacterial activity against *Staphylococcus aureus*, *Bacillus cereus*, and *Escherichia coli*, but showed only marginal activity against *B. cereus* (Table 3). The oils were also screened for *in vitro* cytotoxic activity against MDA-MB-231 human mammary adenocarcinoma and Hs578T human mammary ductal carcinoma cells. The oils did show selective cytotoxicity to MDA-MB-231 cells over Hs578T cells (Table 3). A previous report indicated the leaf essential oil of *Montanoa ovalifolia* from Colombia (composition not reported) had shown marginal cytotoxic activity to Vero and HeLa cells, but no activity against HepG2 or Jurkat cells [31].

## 4. Conclusions

This is the first analysis of the volatiles from *Montanoa guatemalensis*. The leaf oil, rich in cyclocolorenone, α-selinene, and β-selinene, showed selective *in vitro* cytotoxic activity to MDA-MB-231 cells. It is not clear, however, what components are responsible for the cytotoxicity, but the plant may be a good source of cyclocolorenone. Although this work presents preliminary results, it should serve as a template for further experimentation on the leaf oil compositions of *M. guatemalensis*, seasonal, individual, and year-to-year variations.
